# Experimental evidence for the impact of soil viruses on carbon cycling during surface plant litter decomposition

**DOI:** 10.1038/s43705-022-00109-4

**Published:** 2022-03-16

**Authors:** Michaeline B. N. Albright, La Verne Gallegos-Graves, Kelli L. Feeser, Kyana Montoya, Joanne B. Emerson, Migun Shakya, John Dunbar

**Affiliations:** 1 Bioscience Division, Los Alamos National Laboratory, Los Alamos, NM US; 2 Department of Plant Pathology, University of California, Davis, Davis, CA US; 3 Present Address: Allonnia LLC, Boston, MA US

**Keywords:** Microbial ecology, Microbial ecology

## Abstract

To date, the potential impact of viral communities on biogeochemical cycles in soil has largely been inferred from correlational evidence, such as virus-driven changes in microbial abundances, viral auxiliary metabolic genes, and links with soil physiochemical properties. To more directly test the impact of soil viruses on carbon cycling during plant litter decomposition, we added concentrated viral community suspensions to complex litter decomposer communities in 40-day microcosm experiments. Microbial communities from two New Mexico alpine soils, Pajarito (PJ) and Santa Fe (SF), were inoculated onto grass litter on sand, and three treatments were applied in triplicate to each set of microcosms: addition of buffer (no added virus), live virus (+virus), or killed-virus (+killed-virus) fractions extracted from the same soil. Significant differences in respiration were observed between the +virus and +killed-virus treatments in the PJ, but not the SF microcosms. Bacterial and fungal community composition differed significantly by treatment in both PJ and SF microcosms. Combining data across both soils, viral addition altered links between bacterial and fungal diversity, dissolved organic carbon and total nitrogen. Overall, we demonstrate that increasing viral pressure in complex microbial communities can impact terrestrial biogeochemical cycling but is context-dependent.

## Introduction

Viruses that infect microbial hosts have a major impact on their immediate host and can also influence larger scale environmental processes. Viruses account for an estimated 20–40% of bacterial mortality in the oceans and are believed to be a major driver in marine carbon (C) cycling [1–4]. Viral infection likely impacts C and nutrient cycling in aquatic systems [5], and several pathways related to broad-scale impacts on the marine carbon cycle have been proposed [6, 7]. The ‘viral shunt’ emphasizes the recycling of dissolved organic matter (OM), ultimately fueling CO_2_ release into the atmosphere by heterotrophic microbes [8, 9], while the ‘viral shuttle’ emphasizes viral driven organic particle aggregation that favors carbon export to the deep ocean [10].

As in marine systems, recent research suggests that viral-mediated processes also impact terrestrial C and nutrient cycling [11–14]. However, the magnitude and nature of these impacts are unknown. Differences between marine and terrestrial ecosystems that may influence the impact of viral-mediated C cycling include differences in microbe and virus turnover times and the environmental spatial structure and heterogeneity [14, 15]. Efforts to characterize viral diversity [16–21] and activity [22–24] in soils and their impacts on biogeochemical cycling [25] are rapidly increasing. Soil virus studies have focused on exploiting previously sequenced metagenomic [26] and metatranscriptomic [12] datasets as well as creating novel viromics datasets [27]. To date, the impact of the soil virome on biogeochemical cycling has largely been inferred from viral driven changes in microbial populations (e.g., predator-prey cycles) (e.g. [24, 28]) and presence of putative auxiliary metabolic genes, which may be impacting biogeochemical cycling [11, 13, 22, 23]. More direct evidence of soil viral community-mediated alterations in C cycling are generally lacking, but there is recent experimental evidence for soil viruses affecting nitrogen cycling [25]. Given that terrestrial systems contribute approximately 50% of C efflux to the atmosphere [29], understanding the impact of soil viruses may be critical to enable better modeling of greenhouse gas emissions under climate change scenarios.

Changes in the composition of microbial communities are increasingly recognized as a factor that can drive substantial variation in soil carbon cycling [30–35]. Viral predation in litter decomposer communities is a mechanism that can not only alter community composition but also directly affect C flow [15, 36, 37]. To assess the relevance of viral predation, we manipulated virus abundance in microcosm experiments. We inoculated two distinct soil microbial communities spiked with a live or killed viral community concentrate extracted from the same source soil into microcosms containing plant litter and sand substrate; a no virus treatment where buffer was added was used as an additional control (Fig. S1). We measured respiration and net loss of dissolved organic carbon (DOC) and total nitrogen (TN) in the microcosms over 40 days. We measured bacterial and fungal community composition at the 40-day endpoint. Finally, we sequenced the ‘viromes’ and metagenomes of the two initial soil microbial communities. We hypothesized that increasing the quantity of virus would alter microbially driven C cycling during plant litter decomposition by changing microbial community dynamics.

## Methods

### Soil collection, whole microbial community inoculum, and virus extraction

Soils were collected from two locations, Pajarito Mountain Los Alamos (PJ) (35.894208°N, 106.391817°W) and Mount Baldy Santa Fe (SF) (35.793527°N, 105.800391°W) in northern New Mexico during the first week of November in 2020. These sites are at elevations of 2853 m and 2952 m respectively and dominated by pondersa pine and grasses, however, soil samples were collected from areas of bare soil away from immediate vegetation. Larger rocks and leaf litter on top of the soils were removed and soils were aseptically collected with sterilized instruments from an area of approximately 15 × 15 × 15 cm. Samples were transported in a cooler back to the laboratory where they were homogenized and sieved with a 2 mm sieve. Soils were stored for up to one month at 4 °C prior to use in microcosm experiments and we acknowledge that the microbial community could have changed during the storage period.

For each soil type, we used 1 g of the previously sieved and homogenized soil to create a whole community microbial inoculum. To make the microbial inoculum (50× soil dilution) we added 1 g of soil to 9 ml of M9 minimal media, hereafter referred to as M9 media. M9 media was made in quantities of 1 L by combining 778 ml Sterile H_2_0, 200 ml of a well-mixed 5x M9 salts solution (Sigma Aldrich), 1 ml of sterile 1 M MgSO_4_, 0.1 ml of sterile CaCl_2_, and 20 ml of 20% glucose. We shook the soil suspension and then let it sit for 2 min to allow particles to settle particles. We then added 1 ml of the suspension to 4 ml of M9 media.

To concentrate and extract a viral fraction from each soil we used a combination of centrifugation and size filtering to exclude larger microbes. We extracted viruses from 300 g of the two soil types. Fifty grams of soil was added to 10 ml of M9 media and incubated overnight at 37 °C. This was replicated six times for each soil. After the overnight incubation, we further split each sample to allow for centrifugation in the following steps. Twenty-two grams of soil slurry (soil and M9 media) was placed into twelve 50 ml falcon tubes. We added 26 ml of Glycine buffer (0.25 N pH 9.5 – Tween 80 0.02% v/v) to each tube and incubated them at room temperature (RT, 25 °C) for one hour while rotating. Samples were centrifuged at 4500 × *g* for 15 min at 4 °C and the supernatant was collected. The supernatant was then filtered using a 0.22 µM PVDF syringe Millipore filter. After filtering, we used Amicon Ultra filters to concentrate the sample. First, 50 ml Amicon Ultra filters were coated with M9 media to reduce virus adherence to the membrane. This was achieved by adding 1 mL of M9 media to basket, pipetting up and down, and then removing the M9 media. Next, we added 15 ml of our filtered samples and centrifuged at 3000 rpm for 20 min (25 °C). Liquid flow through was discarded and we repeated this step until all of the samples were added to the Amicon filter and approximately 200–400 µL of liquid (concentrated virus) remained in the upper part of the tube. We add 100 µL of M9 media and pipette up and down to mix and resuspend the virus from the Amicon filter. Overall, after pooling samples, we obtained 8 ml of viral concentrate per initial 300 g of soil. An aliquot of virus concentrate from each soil was autoclaved to create a “killed-virus” sample.

In order to test the efficiency of our virus extraction technique, we created control samples for each soil where lambda phage (ATCC 97537) was spiked into a soil slurry (soil and M9 media) and extracted using the method outlined above. For these controls, soil slurries were not incubated overnight. Following the phage extraction, we performed plaque assays to assess extraction efficiency. Lambda phage susceptible *Escherichia coli* (ATCC 47076) were grown overnight in lambda broth mixing on a rotator at 37 °C. We added 0.3 ml of *E.coli* and 0.1 ml of phage sample and mixed in a 2 ml tube. Phage samples included extractions from the two lambda spiked soils, a control of the initial lambda phage that was spiked into soils, and lambda phage that had been autoclaved to kill the phage. Serial dilutions of each sample type were tested and an *E. coli* control with no phage addition was included. After mixing the *E. coli* and phage, we incubated tubes at RT (25 °C) for 20 min and then moved tubes to a heat-block where they were incubated at 37 °C for 10 min. 0.4 ml of *E.coli* and phage mix was then added to 2.5 ml of heated lambda top agar and poured on pre-prepared lambda plates. Plates were placed in the incubator at 37 °C overnight and plaques were counted on each plate and used to compute the concentration of viable lambda phage in each sample.

### Initial soil total microbial community and viral DNA extraction for metagenomic sequencing

Bulk microbial and viral extractions from soils were completed within a week following soil collection, following a protocol modified from Göller et al. [38] and Thurber et al. [39]. A protein supplemented phosphate-buffered saline (PPBS) elution buffer was created in 1 L quantities by combining 10 mL of 10× phosphate-buffered saline (Fisher Scientific), 10 g of monohydrate potassium citrate (Spectrum Chemical), 18.05 g of anhydrous MgSO_4_ (Fisher Scientific) and enough sterile water to reach 1 L. The pH was adjusted to 6.5 and 20 g of nuclease- and protease-free bovine serum albumin (“BSA”, VWR) was slowly added while the solution was mixing using a stir plate. Once the BSA was solubilized, the solution was filtered through a 0.22 µm vacuum filter and stored at 4 °C. Soil was sieved at 2-mm and 30 g was weighed out for each soil, split into two 50 mL Falcon tubes. Soils were suspended in a 1:1 w/v PPBS elution buffer, the Falcon tubes were parafilmed and shaken manually for 30 s and vortexed for 30 s in sequence four times. Samples were then shaken at 300 RPM for 40 min at RT and then soil suspensions were refrigerated overnight at 4 °C. The following day, samples were manually shaken again for 2 min and were centrifuged of 5 min at 5k RPM at 4 °C. Supernatant was removed, decanted and filtered through sterile fine mesh (0.5 mm) to remove floating debris and then saved. The pellet was then resuspended in 1:1 w/v PPBS buffer, using sterile scoopulas to physically loosen and mix the soil pellet in the liquid. Samples were shaken for 30 min at 300 rpm at RT and then centrifuged for five minutes at 5k RPM at 4 °C. Supernatant collected and pellet resuspension was completed a total of three times. Supernatants were combined and centrifuged three times for 10 min at 5k RPM at 4 °C. Following each centrifugation supernatant was transferred to a new tube and the pellet was discarded. The liquid was then filtered sequentially through a 25 µm cellulose filter (Cytiva Whatman™ Qualitative Filter Paper: Grade 4 Circles, Fisher Scientific), 11 µm filter paper (Cytiva Whatman™ Qualitative Filter Paper: Grade 1 Circles, Fisher Scientific), 3 µm, (Cytiva Whatman™ Qualitative Filter Paper: Grade 6 Circles, Fisher Scientific), 1 µm (Cytiva Qualitative Grade Plain Filter Paper Circles - P5 Grade, Fisher Scientific), 0.45 µm (Fisherbrand™ Disposable PES Filter Units, Fisher Scientific), and finally 0.22 µm syringe-driven PES filters (MilliporeSigma). Note that 550 µL aliquots of the 11 µm filtrate was reserved for the bulk community DNA extraction. Liquid was stored overnight at 4 °C. Sample volumes of the viral-enriched 0.22 µm filtrate ranged from approximately 40–50 ml. Viral concentration was achieved in two rounds. First, 25 ml of the 0.22 µm filtrate and 10 µL of 0.02 µm filtered FeCl_3_ (with a concentration of 0.1 g/mL) were added to sterile 26.3 ml polycarbonate tubes and ultracentrifuged for 3 h at 35,000 RPM under a vacuum. Tubes were carefully decanted reserving the supernatant. The remaining filtrate was added to the same ultracentrifuge tube, along with enough of the reserved supernatant from the previous round, if needed, to create a total volume of 25 ml. 10 µL of the FeCl_3_ solution was again added and the tubes were centrifuged again under the same conditions. Tubes were decanted and 400 µL of ultra-pure water was added to resuspend the pellets, which were allowed to further dissolve overnight at 4 °C. After overnight incubation, remaining pellet material as mixed with sterile scoopulas and pipetted into a new tube. 200 µL of ultra-pure water was added and further mixing was performed with scoopulas and pipetting liquid up and down. The final elution volume was approximately 600 µL. Viral-enriched samples were then treated with 15 µL RNase-free DNase (Qiagen, Cat No. 79254) and 60 µL of the accompanying buffer and incubated at RT on a shaker at 600 RPM for 15 min. Tubes were removed to manually invert every few minutes. DNase was inactivated by adding 10 mmol EDTA and incubating for 10 min at 65 °C. Samples were then cooled and stored at 4 °C until extraction. Both the bulk community samples (550 µL of the 11 µm filtrate) and the viral-enriched samples were extracted within 48 h. Samples were extracted using DNeasy PowerSoil Kits (Qiagen, Germantown, MD) using the full volume of sample available and following manufacturer instructions. At the final step, samples were eluted twice in 100 µL of ultra-pure water (viral) or twice in 50 µL of ultra-pure water (bulk community). The concentration of DNA was obtained using the Qubit DNA Assay Kit (ThermoFisher Scientific, Cat. # Q32854). The quality of the DNA was determined by running the sample on an E-Gel 1% agarose gel (ThermoFisher, Cat# G402001) with Lambda DNA/HindIII Marker (ThermoFisherScientific, Cat. #FER SM0103).

Illumina libraries were prepared using NEBNext Ultra DNA II Library Preparation Kit (New England Biolabs, Cat. #E7645L). DNA was fragmented using a Covaris E220, the ends made blunt and adapters and indexes were added onto the ends of the fragments to generate Illumina libraries that could be sequenced on an Illumina sequencer. Illumina libraries were eluted in DNA Elution Buffer (Zymo Research, Cat. #D3004-4-10). The concentration and size of the libraries was determined by the Agilent D5000 Assay (Agilent, Cat. #5067-5588, 5067-5589). An accurate library quantification was determined using the Library Quantification Kit – Illumina/Universal Kit (KAPA Biosystems, KK4824). Libraries were normalized to the same concentration based on the qPCR results. The libraries were sequenced on the Illumina NextSeq generating paired-end 151 bp reads using the NextSeq 500/550 High Output Kit v2.5 (300 cycles) (Illumina, Cat. #20024908). Sequence data are available through MG-RAST (www.mgrast.org, mgp100914).

### Metagenomic sequence processing

Raw metagenomic and metaviromic reads were quality controlled using FaQCs v2.10 with default parameters [40]. Cleaned reads were then assembled to contigs using SPAdes v3.13.0 with the *--meta* flag [41, 42]. Contigs that were at least 1 kb of length and had 4 ORFs were further processed using VIBRANT v1.2.1 to identify viral contigs [43], using the default settings for metagenome contigs and the viral decontamination *-virome* flag on the metavirome contigs. Cleaned raw reads were then mapped back to all viral contigs using BWA mem v0.7.17-r1188 and then the consequent TPMs (Transcript Per Million) and RPKMs (Read Per Kilo Million) for contigs were calculated using a custom script. Viral contigs were further quality controlled using CheckV v0.8.1 [44] and then clustered into vOTUs using scripts described previously in [45]. Viral contigs were then assigned to taxonomy based on the Last common ancestor (LCA) of all the CDS in a contig. Briefly, CDSs for each viral contigs were predicted using phanotate v1.5.0 [46], which were then searched against viral proteins from GenBank viral genome database (last accessed on August 7, 2021) using diamond v0.9.14.115 [47]. Only hits that had e-value greater than 10e-4 and at least 50% of both query and target sequence covered were kept. LCA of the taxonomy of the top hit for each CDS were then assigned as the taxonomy of the contig.

### Microcosm experiment

Microcosms were constructed in 125 ml serum bottles with approximately 7 g of sand and 0.1 g (dry weight) of *Blue grama* grass litter cut into 1 cm pieces. The microcosms were sterilized by autoclaving (at 121 °C and 15 psi) three times for 1 h each, with at least a 12-h resting interval between each autoclave cycle.

For each of the two soil types, we had three different treatments, these included (1) whole microbial community (no added virus), (2) whole microbial community and killed-virus extraction (+killed-virus), and (3) whole microbial community and virus extraction (+virus) (Fig. S1) with three replicates per treatment type for a total of 18 microcosms. The whole microbial community and virus extractions treatments were always from the same soil. We first created a homogenized inoculum for each treatment by adding viral extract to whole microbial inoculum at a ratio of 2:1. For the no added virus treatment we added M9 media. We then added 1.5 ml of homogenized inoculum to each sterilized grass litter and sand microcosm. Microcosms were sealed with Teflon-lined crimp caps (preventing desiccation) and incubated at 25 °C with a 12-h light–dark cycle for 41 days. On days 2, 5, 8, 14, 21, 32, and 41, CO_2_ was measured by gas chromatography using an Agilent Technologies 490 Micro GC (Santa Clara, CA, USA). After each measurement, the headspace air was evacuated with a vacuum pump and replaced with sterile-filtered air.

After the 41-day incubation, microcosms were destructively sampled to measure DOC, TN, and community composition. For each microcosm, 7 ml of sterile deionized water was added, swirled gently by hand for 30 s and then filtered through a 0.2 µm filter and stored at −20 °C until TOC analysis. The remaining sand, plant litter and water mixture from each microcosm was frozen at −80 °C for DNA extraction. In addition, to measuring DOC and TN in the final microcosms, initial concentrations from the blue grama plant litter, M9 media, and viral concentrate for PJ and SF soils were also measured. All samples were prepped by adding 1 mL of sample to 5 N HCl to acidify and purged with air to remove inorganic carbon. The DOC and TN concentration of each sample was then measured on an OI Analytical model 1010 wet oxidation TOC analyzer (Xylem Inc., Rye Brook, NJ, USA). Net loss of DOC and TN from each microcosm was measured by subtracting the final microcosm measurements from the initial concentrations.

### Microbial community taxonomic profiling

For the final microcosm samples, we extracted and sequenced DNA to obtain bacterial (16S rRNA) and fungal (ITS) community profiles. DNA extractions were performed with a DNeasy PowerSoil Kit (Qiagen, Hilden, Germany) following the manufactures protocol with the following exceptions, 0.3 g of material was used per sample extract and all samples were eluted to a final volume of 30 µl. DNA samples were quantified with the Invitrogen Qubit dsDNA HS Assay Kit (Invitrogen,Waltham, MA), on an Invitrogen Qubit 2.0 following the manufacturers protocols.

The bacterial 16S rRNA genes were amplified with methods previously described in [35] with the following modifications. Following step one of PCR the PCR products were cleaned with a 0.9 Ratio of Beckman Coulter Agentcourt AMPure XP beads (Beckman Coulter, Brea, CA) followed by step two of PCR. Amplicons were cleaned using the same method as the PCR1 products and quantified using the same procedure as the extracted DNA, then pooled to 10 ng each. The pool was then cleaned with beads in the same manner as above following manufacturers protocol. The fungal ITS regions were amplified using an equimolar mixture of three ITS9 forward primers (ITS9f_FS1: TCGTCGGCAGCGTCAGATGTGTATAAGAGACAGNNNNNNGAACGCAGCRAAIIGYGA, ITS9f_FS2: TCGTCGGCAGCGTCAGATGTGTATAAGAGACAGNNNNNGAACGCAGCRAAIIGYGA, and ITS9F_FS3: TCGTCGGCAGCGTCAGATGTGTATAAGAGACAGNNNNGAACGCAGCRAAIIGYGA) and the ITS4r_FS reverse primer (GTCTCGTGGGCTCGGAGATGTGTATAAGAGACAGNNNNNNTCCTCCGCTTATTGATATGC) [48]. Amplification procedure was used based on Gloor et al. [49], with Phusion Hot Start II High Fidelity DNA polymerase (Thermo Fisher Scientific, Vilnius, Lithuania). In the first PCR, barcoded amplicons were produced over 25 cycles using gene primers flanked by 6 nt barcodes that jointly provided a unique 12-mer barcode for each sample [49]. Cycling conditions were 5 min at 95 °C, 25 cycles of (95 °C for 30 s, 50 °C for 60 s, 68 °C for 60 s), and a final extension step of 68 °C for 10 min. The second PCR extended Illumina adapter sequences on the amplicons over 12 cycles. Cycling conditions were 5 min at 95 °C, 12 cycles of (95 °C for 30 s, 50 °C for 60 s, 68 °C for 60 s), and a final extension step of 68 °C for 10 min. Amplicons were cleaned and concentrated using the same procedure as with the bacterial 16S rRNA genes described above. DNA quality of the bacterial and fungal amplicon pools were assessed with a bioanalyzer, concentration was verified by qPCR, and sequencing was performed on an Illumina MiSeq with paired-end 250 bp chemistry at Los Alamos National Laboratory. Unprocessed sequence data are available through NCBI’s Sequence Read Archive (PRJNA763874)

Sequence data were processed using UPARSE [50], where the same methods as previously described in [51] where used to obtain OTU tables. Post processing, for bacteria we retained 83,903 reads; per sample OTU count minimum, maximum, and median were 1560, 7950, and 4661 reads respectively. Post processing, for fungi we retained 81,674 reads; per sample OTU count minimum, maximum, and median were 2043, 8006, and 4804 reads respectively. OTU tables were rarefied to the lowest number of common sequences for bacterial and fungal profiles (*n* = 1560 and *n* = 2043, respectively). OTU tables were used to calculate Bray-Curtis distance matrices and diversity metrics (richness and Shannon diversity) [52].

### Statistical analysis

Many of the statistical tests were performed separately for each microbial inoculum (PJ and SF) as we expected substantial differences in the microbial composition and nutrient inputs across the different inocula due to the geographic distance of the initial soil collection points and wanted to focus on differences across treatments. All statistical analyses and graphing were performed in the R software environment [53]. To test for differences in the net loss of DOC and TN among treatments in the PJ and SF microcosms over 41 days we performed ANOVA analyses (aov function, R). We also used ANOVAs to test the impacts of treatment on CO_2_ production at each measured time point. To test for differences in bacterial and fungal composition among treatments we performed PERMANOVA analyses (vegan package R [52]). To identify taxa contributing to significant compositional differences, we used indicator species analysis [54]. Lastly, we combined data across both soils and for each treatment (no added virus, +killed-virus, +virus) and we tested for correlations between bacterial and fungal richness and diversity and carbon and nutrient measurements. Here we used DOC and TN measurements from the final microcosm sampling which corresponds to the sampling time for the microbial community data. For this analysis, we used the Hmisc [55] and corrr [56] R packages using Spearman’s rho rank correlation [57]. Correlations were visualized as networks using qgraph [58].

## Results and discussion

### Virus extraction efficiency and magnitude of virus additions

We used lambda phage additions to soils to test the efficiency of our virus extraction protocol and used this to get an estimate of the magnitude of viral addition in our +virus treatment compared to the controls. Based on plaque assays, our soil virus extraction procedure recovered the lambda phage from soil with at least 50% efficiency (Fig. S2). Given this, based on the amount of soil from which our virus concentrates were generated, we estimated that our virus addition treatment in microcosms increased virus abundance over 15-fold. Of course, recovery of soil viruses may be slightly lower since extraction efficiency can vary among viruses owing to differential binding to filters during the extraction protocol [59]. While our virus addition is artificial, large increases in virus abundance in ecosystems are expected when viral lytic cycles are triggered [60].

### Viral additions impact ecosystem processes

The impact of virus addition was assessed by comparing the +virus treatment to two controls: no added virus and +killed-virus (Fig. S1). Based on visual inspection (e.g., the presence of dark material in the viral concentrate), our soil virus concentrates from both soils contained additional OM, so the +virus treatment inevitably boosted nutrient abundance in addition to viral abundance in the microcosms. This can also be seen in the inoculum DOC and TN measurements, which were similar in the + killed-virus treatment and the +virus treatment and both were much higher than the no added virus treatment (Fig. S3). Thus, we used the +killed-virus treatment to assess the impact of the large carbon and nutrient addition on the system (Fig. S1). We acknowledge that, in addition to ‘killing’ the viruses, autoclaving changes the character of carbon and nutrients [61], which is a limitation to the study. We attempted to mitigate this by comparing the +virus treatment to two different controls (no added virus and +killed-virus treatments). Other studies manipulating viruses in complex soil systems faced similar limitations [25].

**Fig. 1:**
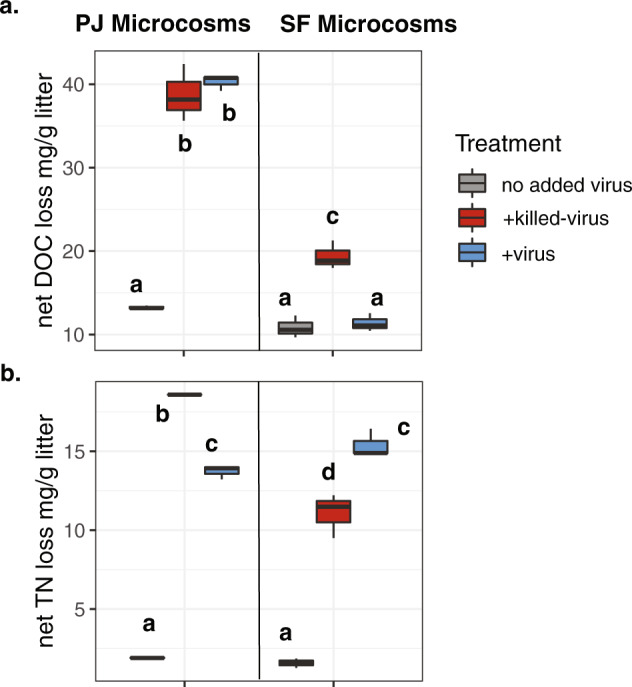
Viral addition impacts carbon and nutrient cycling. Net loss of **a** DOC and **b** TN in the PJ and SF microcosms. Letters indicate significant differences across treatments and soil inoculum (Tukey hsd posthoc test).

We focused on two metrics of C cycling in the decomposition microcosms including net DOC loss and respiration (CO_2_ production). Comparing across treatments within the PJ and SF microcosms, the PJ +virus treatment had the same net DOC loss compared to the +killed-virus treatment, while for the SF microcosms net DOC loss was lower in the +virus compared to the +killed-virus treatments (Fig. 1a). We also measured net TN loss in the system and found that the PJ +virus treatment had a lower net TN loss compared to the +killed-virus treatment, while in the SF microcosms we saw the opposite trend (Fig. 1b). Net DOC loss captures the net outcome of microbial consumption and production of DOC in the microcosm system. Generally, consumption was much higher than production, illustrated by the overall decline in DOC from the beginning to end of the experiment (Fig. S3). We posited that viruses would alter the net DOC measured in the system by changing microbial community dynamics. For example, increasing phage lysis of an abundant microbial taxa might open niche space and increase competition among other taxa leading to altered DOC consumption. Thus, our hypothesis was partially supported as viral addition altered net DOC loss, one component of C cycling in this system, in the SF microcosms. Furthermore, nutrient cycling, specifically net TN loss, was altered in both the SF and PJ microcosms, but the direction of the effect was community specific. Previous work looking at the effects of virus additions on inorganic N in soils, using a similar autoclaved (+killed-virus) and non-autoclaved (+virus) experimental design, also found significant impacts of virus addition on N cycling in some soil microbial communities, but not others [25].

**Fig. 2:**
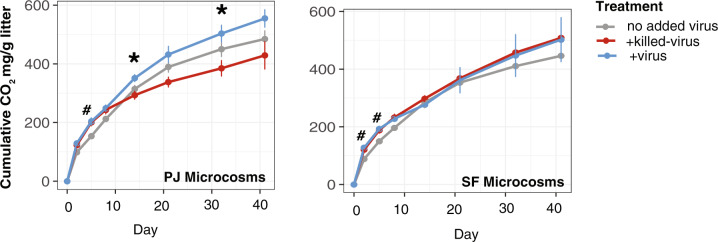
Viral addition impacts respiration. Cumulative respiration across the treatments over 40 days for PJ (left) and SF (right) inoculated microcosms. Significant differences (*p* < 0.05) between the no added virus and the virus addition (+virus and +killed-virus) treatments are denoted with the symbol (#). Significant differences (*p* < 0.05) between the +virus and +virus-killed treatments are denoted with the symbol (*).

The other component of C cycling that we assessed in our system was respiration; here we also observed viral impacts. Generally, microcosm respiration dynamics followed a monotonic decline that is consistent with a fixed-carbon pools and first-order growth kinetics [62]. In the early stages, the microbes inoculated into the system have lots of space and resources to expand into and so they experience rapid growth which then levels off, due to a decline in easily accessible resources and space as the microcosms reach a carrying capacity. Initially, over the first eight days post inoculation, respiration was higher in both the +virus and +killed-virus treatment compared to the control treatment in both the PJ and SF soil (Fig. 2). This initial high respiration was likely driven by the OM addition described above. In the PJ microcosms by day 14, respiration was significantly higher in the +virus treatment compared to the +killed-virus treatment and differences between these treatments increased to 30% by day 41 (Fig. 2). By contrast, in the SF microcosms after the initial surge in respiration in the +virus and +killed-virus compared to the no added virus treatment, by day 14 respiration in all samples was similar, and this persisted for the remaining duration of the experiment (Fig. 2). Our hypothesis was partially supported; in one microbial community, increasing virus quantity altered respiration, while in the other microbial community, it did not.

We posit that differences in the abundance and/or diversity of viruses in the original soils and thus in the viral concentrates may have led to these microbial community-specific responses in both respiration and DOC abundance. In support of this, bulk metagenomic sequencing data from original soils showed a 10-fold higher abundance of viral reads in the PJ (0.03%) compared to the SF (0.003%) soils (Table S1). We expect these initial differences would propagate to the viral concentrates and thus could impact magnitude of treatment effects from the viral spike-ins. Furthermore, in the viral-enriched metagenomes from the original soils many more unique viruses were identified in the PJ (68,329 vOTUs) compared to the SF (6,735 vOTUs) soil and viral diversity differed across the two soil samples (Table S2). Disentangling the relative impact of these two aspects, abundance and diversity, of viruses on ecosystem functioning requires further inquiry. Reliable viral counts from soil can be difficult to obtain, for example due to non-specific binding of epifluorescence dyes to soil particles in addition to viral particles [63], but robust measurements of viral biomass could add rigor to future studies. Furthermore, given the high spatial variation observed in viral communities in soils in a previous study [27], future work is needed to assess the likelihood of viral impacts on soil C cycling across distinct communities and soil types to better assess the magnitude of potential ecosystem scale impacts.

### Viral addition impacts bacterial and fungal composition

**Fig. 3:**
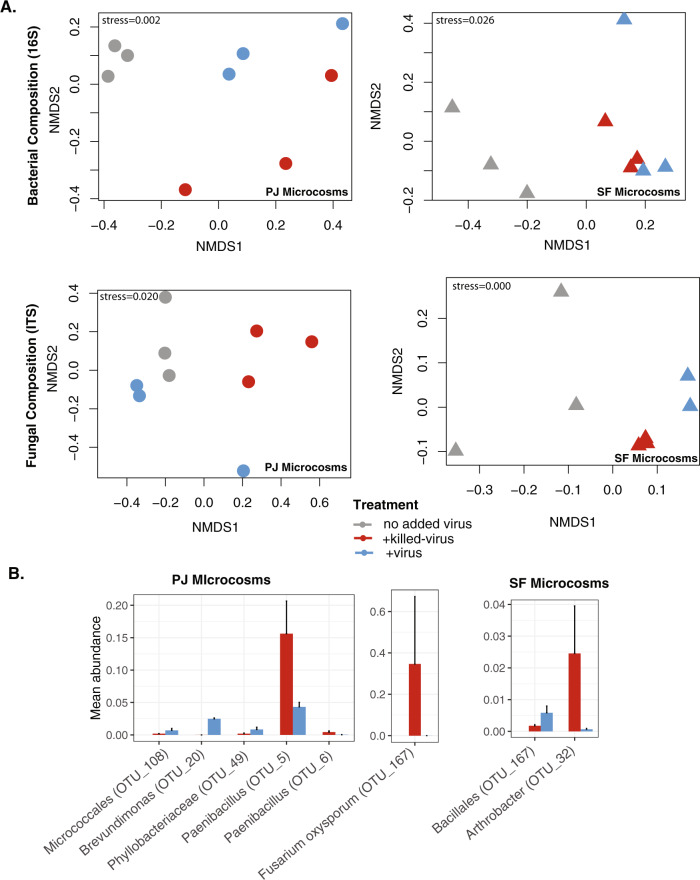
Viral addition impacts microbial composition. **A** Bacterial (top) and fungal (bottom) composition in PJ (left) and SF (right) inoculated microcosms. **B** Mean relative abundance of taxa showing significant differences across the +killed-virus and +virus treatments identified through indicator species analysis [54].

As we hypothesized, virus additions significantly altered microbial community composition. We observed significant differences across treatment groups (no added virus, +virus, +killed-virus) for bacterial and fungal composition in the PJ and SF microcosms, respectively (Fig. 3A, Table S3). Specifically, in the +killed-virus compared to the +virus treatment two OTUs in the bacterial genus *Paenibacillus* and one OTU belonging to the fungal species *Fusarium oxysporum* significantly increased in relative abundance in the PJ microcosms, and one *Arthrobacter* OTU significantly increased in relative abundance in the SF microcosms (Fig. 3B, Table S4). Many bacteriophages/mycoviruses are known to be active against bacterial/fungal strains of the genera *Paenibacillus* [64], *Arthrobacter* [65] and *Fusarium* [66]. We posit that these taxa were impacted by predation in the +virus treatment, reducing their relative abundance. Although mycoviruses are generally thought to be obligately intracellular and are not known to exist as free viral particles in soil, very limited data exist on viruses of soil fungi [67]. In addition, increased viral predation on a few key taxa may have cascading impacts on overall microbial community assembly and successional processes during plant litter decomposition. In terms of their role in the decomposition process, both bacterial taxa *Paenibacillus* and *Arthrobacter* are known for cellulolytic ability [68, 69], and they and the fungus *Fusarium*, a known lignin degrader [70], have been reported as keystone litter decomposer taxa [71]. Thus, changes in the relative abundance of these taxa may impact plant litter decomposition.

Many taxa also showed differences in abundance between the no added virus and the other treatment groups (Fig. S4, Table S4). This was primarily due to the presence of additional taxa in the no added virus treatment. Overall, the richness and Shannon diversity of bacteria and fungi in the +virus and +killed-virus treatments were reduced compared to the no added virus treatment in both PJ and SF microcosms (Fig. S5). Nutrient addition including OM residuals from the viral concentration and extraction from soil may have contributed to this as nutrient addition often reduces microbial richness [72]. In the PJ microcosms we saw a trend of increased bacterial and decreased fungal richness and Shannon diversity in the +virus compared to the +killed-virus and the opposite trend in the SF microcosms, but it was not significant in either case (Fig. S5).

### Viral addition alters links between microbial community traits and ecosystem processes

**Fig. 4:**
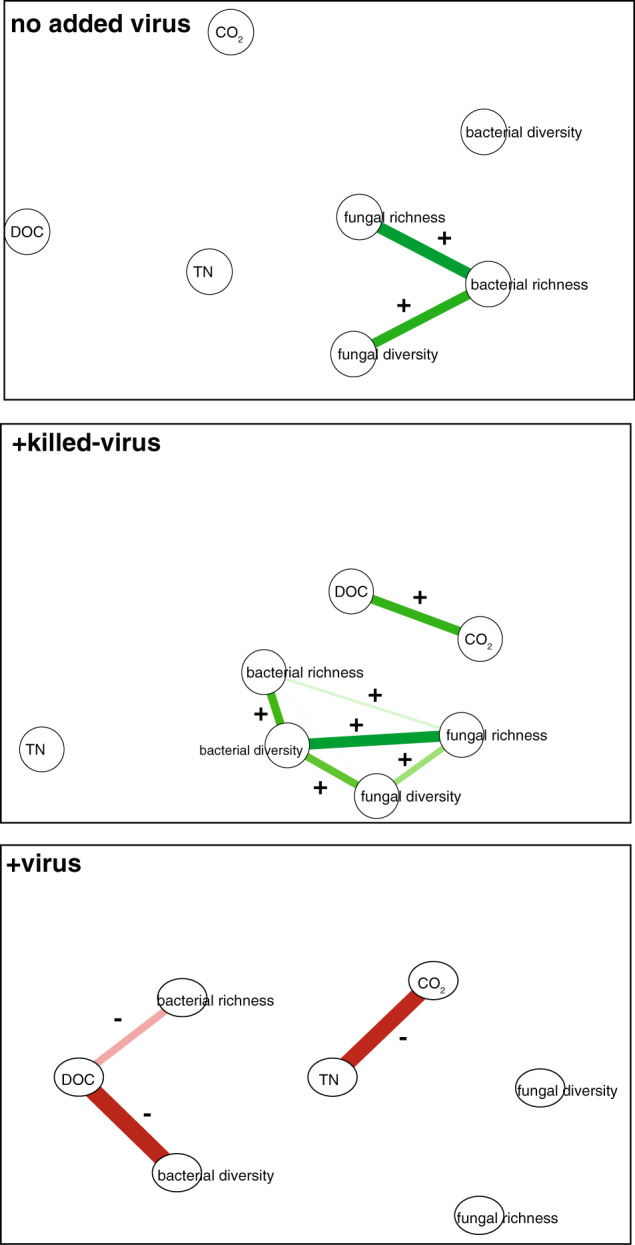
Viral addition alters correlations between microbial community traits and ecosystem processes. Spearman’s rho rank correlations (*p* > 0.05) between bacterial and fungal richness and diversity and carbon and nutrient measurements are shown. Thicker lines indicate increasing r values. (See Fig. S6 for a correlation heatmap with more detailed information about correlations across all variables).

To test the impact of viral addition on links between the microbial community and ecosystem processes, we aggregated carbon, nutrient, and microbial community trait (bacterial and fungal diversity) metrics across both soil types for each of the three treatments. This analysis provided additional support of our hypothesis, where viral addition altered the relationship between carbon and nutrients and microbial community traits (Fig. 4, Fig. S6). In our two control treatments, we saw positive correlations between bacterial and fungal diversity, which were lost in our +virus treatment. In the +virus treatment, we saw a negative correlation between DOC and bacterial diversity, which was not observed in the two control treatments. This is further evidence that manipulating the viral community has cascading impacts on microbial community assembly during plant litter decomposition. Impacts of polymicrobial (e.g. bacterial-fungal-virus) interactions on ecosystem functioning has been suggested in other systems [73, 74]. Furthermore, in our study, it was also interesting to note that the +virus outcomes were not simply an additive result of nutrient addition (+killed-virus) and predation. This is another illustration of how viral impacts on ecosystem functioning may be context dependent, in this case based on nutrient availability.

## Conclusions

While previous studies have shown correlations between viral abundance and carbon compounds [2] and used meta-omics to infer functional impacts of viruses on C cycling [5, 11, 13, 20, 21, 23, 75, 76], here we experimentally demonstrated impacts of soil viruses on carbon cycling by manipulating virus abundance in complex microbial communities. Our results show that increases in soil virus abundance can impact carbon and nutrient cycling in terrestrial systems likely by altering microbial community dynamics. However, the magnitude of these effects depended on factors such as community composition and nutrient availability. Future research is needed to delve into virus-host spatial and temporal dynamics in soils, where the physical structure may change dynamics compared to a more well-mixed marine ecosystem.

## Supplementary information

Table S1

Table S2

Table S3

Table S4

Supplementary Figure Legends

Figure S1

Figure S2

Figure S3

Figure S4

Figure S5

Figure S6

## References

[1] Brum JR, Sullivan MB (2015). Rising to the challenge: accelerated pace of discovery transforms marine virology. Nat Rev Microbiol..

[2] Danovaro R, Corinaldesi C, Dell’Anno A, Fuhrman JA, Middelburg JJ, Noble RT (2011). Marine viruses and global climate change. Fems Microbiol Rev..

[3] Suttle CA (2007). Marine viruses - major players in the global ecosystem. Nat Rev Microbiol..

[4] Suttle CA (2005). Viruses in the sea. Nature..

[5] Guidi L, Chaffron S, Bittner L, Eveillard D, Larhlimi A, Roux S (2016). Plankton networks driving carbon export in the oligotrophic ocean. Nature..

[6] Zimmerman AE, Howard-Varona C, Needham DM, John SG, Worden AZ, Sullivan MB (2020). Metabolic and biogeochemical consequences of viral infection in aquatic ecosystems. Nat Rev Microbiol..

[7] Peduzzi P, Weinbauer MG (1993). Effect of Concentrating the Virus-Rich 2-200-Nm Size Fraction of Seawater on the Formation of Algal Flocs (Marine Snow). Limnol Oceanogr..

[8] Wilhelm SW, Suttle CA (1999). Viruses and Nutrient Cycles in the Sea - Viruses play critical roles in the structure and function of aquatic food webs. Bioscience..

[9] Fuhrman JA (1999). Marine viruses and their biogeochemical and ecological effects. Nature..

[10] Sullivan MB, Weitz JS, Wilhelm S (2017). Viral ecology comes of age. Env Microbiol Rep..

[11] Emerson JB, Roux S, Brum JR, Bolduc B, Woodcroft BJ, Jang HB (2018). Host-linked soil viral ecology along a permafrost thaw gradient. Nat Microbiol..

[12] Starr EP, Nuccio EE, Pett-Ridge J, Banfield JF, Firestone MK (2019). Metatranscriptomic reconstruction reveals RNA viruses with the potential to shape carbon cycling in soil. Proc Natl Acad Sci USA..

[13] Trubl G, Jang HB, Roux S, Emerson JB, Solonenko N, Vik DR (2018). Soil viruses are underexplored players in ecosystem carbon processing. mSystems..

[14] Williamson KE, Fuhrmann JJ, Wommack KE, Radosevich M (2017). Viruses in soil ecosystems: an unknown quantity within an unexplored territory. Annu Rev Virol..

[15] Emerson JB (2019). Soil viruses: a new hope. mSystems..

[16] Liang XL, Zhang YY, Wommack KE, Wilhelm SW, DeBruyn JM, Sherfy AC (2020). Lysogenic reproductive strategies of viral communities vary with soil depth and are correlated with bacterial diversity. Soil Biol Biochem..

[17] Liang XL, Wang YS, Zhang Y, Zhuang J, Radosevich M (2021). Viral abundance, community structure and correlation with bacterial community in soils of different cover plants. Appl Soil Ecol..

[18] Roy K, Ghosh D, DeBruyn JM, Dasgupta T, Wommack KE, Liang X (2020). Temporal dynamics of soil virus and bacterial populations in agricultural and early plant successional soils. Front. Microbiol..

[19] Williamson KE, Radosevich M, Wommack KE (2005). Abundance and diversity of viruses in six Delaware soils. Appl Environ Microb..

[20] Lee S, Sieradzki ET, Nicolas AM, Walker RL, Firestone MK, Hazard C (2021). Methane-derived carbon flows into host-virus networks at different trophic levels in soil. Proc Natl Acad Sci USA..

[21] ter Horst AM, Santos-Medellin C, Sorensen JW, Zinke LA, Wilson RM, Johnston ER (2021). Minnesota peat viromes reveal terrestrial and aquatic niche partitioning for local and global viral populations. Microbiome..

[22] Wu RN, Davison MR, Gao YQ, Nicora CD, Mcdermott JE, Burnum-Johnson KE (2021). Moisture modulates soil reservoirs of active DNA and RNA viruses. Commun Biol..

[23] Trubl G, Kimbrel J, Liquet-Gonzalez J, Nuccio E, Weber P, Pett-Ridge J (2021). Active virus-host interactions at sub-freezing temperatures in Arctic peat soil. Microbiome..

[24] Van Goethem MW, Swenson TL, Trubl G, Roux S, Northen TR (2019). Characteristics of wetting-induced bacteriophage blooms in biological soil crust. Mbio..

[25] Braga LPP, Spor A, Kot W, Breuil MC, Hansen LH, Setubal JC (2020). Impact of phages on soil bacterial communities and nitrogen availability under different assembly scenarios. Microbiome..

[26] Ren J, Song K, Deng C, Ahlgren NA, Fuhrman JA, Li Y (2020). Identifying viruses from metagenomic data using deep learning. Quant Biol..

[27] Santos-Medellin C, Zinke LA, Ter Horst AM, Gelardi DL, Parikh SJ, Emerson JB (2021). Viromes outperform total metagenomes in revealing the spatiotemporal patterns of agricultural soil viral communities. ISME J..

[28] Srinivasiah S, Lovett J, Ghosh D, Roy K, Fuhrmann JJ, Radosevich M (2015). Dynamics of autochthonous soil viral communities parallels dynamics of host communities under nutrient stimulation. Fems Microbiol Ecol..

[29] Schimel DS, House JI, Hibbard KA, Bousquet P, Ciais P, Peylin P (2001). Recent patterns and mechanisms of carbon exchange by terrestrial ecosystems. Nature..

[30] Glassman SI, Weihe C, Li JH, Albright MBN, Looby CI, Martiny AC (2018). Decomposition responses to climate depend on microbial community composition. Proc Natl Acad Sci USA..

[31] Strickland MS, Lauber C, Fierer N, Bradford MA (2009). Testing the functional significance of microbial community composition. Ecology..

[32] Matulich KL, Martiny JBH (2015). Microbial composition alters the response of litter decomposition to environmental change. Ecology..

[33] Schimel JP, Schaeffer SM (2012). Microbial control over carbon cycling in soil. Front Microbiol..

[34] Anthony MA, Crowther TW, Maynard DS, van den Hoogen J, Averill C (2020). Distinct assembly processes and microbial communities constrain soil organic carbon formation. One Earth..

[35] Albright MBN, Johansen R, Thompson J, Lopez D, Gallegos-Graves LV, Kroeger ME (2020). Soil bacterial and fungal richness forecast patterns of early pine litter decomposition. Front Microbiol..

[36] Kuzyakov Y, Mason-Jones K (2018). Viruses in soil: Nano-scale undead drivers of microbial life, biogeochemical turnover and ecosystem functions. Soil Biol Biochem..

[37] Trubl G, Hyman P, Roux S, Abedon ST (2020). Coming-of-age characterization of soil viruses: a user’s guide to virus isolation, detection within metagenomes, and viromics. Soil Syst..

[38] Goller PC, Haro-Moreno JM, Rodriguez-Valera F, Loessner MJ, Gomez-Sanz E (2020). Uncovering a hidden diversity: optimized protocols for the extraction of dsDNA bacteriophages from soil. Microbiome..

[39] Thurber RV, Haynes M, Breitbart M, Wegley L, Rohwer F (2009). Laboratory procedures to generate viral metagenomes. Nat Protoc..

[40] Lo CC, Chain PSG (2014). Rapid evaluation and quality control of next generation sequencing data with FaQCs. Bmc Bioinform..

[41] Nurk S, Meleshko D, Korobeynikov A, Pevzner PA (2017). metaSPAdes: a new versatile metagenomic assembler. Genome Res..

[42] Prjibelski A, Antipov D, Meleshko D, Lapidus A, Korobeynikov A (2020). Using SPAdes de novo assembler. Current protocols in bioinformatics..

[43] Kieft K, Zhou ZC, Anantharaman K (2020). VIBRANT: automated recovery, annotation and curation of microbial viruses, and evaluation of viral community function from genomic sequences. Microbiome..

[44] Nayfach S, Camargo AP, Schulz F, Eloe-Fadrosh E, Roux S, Kyrpides NC (2021). CheckV assesses the quality and completeness of metagenome-assembled viral genomes. Nat Biotechnol..

[45] Nayfach S, Paez-Espino D, Call L, Low SJ, Sberro H, Ivanova NN (2021). Metagenomic compendium of 189,680 DNA viruses from the human gut microbiome. Nat Microbiol..

[46] McNair K, Zhou C, Dinsdale EA, Souza B, Edwards RA (2019). PHANOTATE: a novel approach to gene identification in phage genomes. Bioinformatics..

[47] Buchfink B, Xie C, Huson DH (2015). Fast and sensitive protein alignment using DIAMOND. Nat. Methods..

[48] de Souza RS, Okura VK, Armanhi JS, Jorrin B, Lozano N, da Silva MJ (2016). Unlocking the bacterial and fungal communities assemblages of sugarcane microbiome. Sci Rep..

[49] Gloor GB, Hummelen R, Macklaim JM, Dickson RJ, Fernandes AD, MacPhee R (2010). Microbiome profiling by illumina sequencing of combinatorial sequencetagged PCR products. PLoS ONE..

[50] Edgar RC (2013). UPARSE: highly accurate OTU sequences from microbial amplicon reads. Nat Methods..

[51] Albright MBN, Sevanto S, Gallegos-Graves L, Dunbar J (2020). Biotic interactions are more important than propagule pressure in microbial community invasions. Mbio..

[52] Oksanen J, Blanchet F, Friendly M, Kindt R, Legendre P, McGlinn D, et al. vegan: Community Ecology Package. 2020. R package version 2.5-7. https://CRAN.Rproject.org/package=vegan

[53] Team RC R: a language and environment for statistical computing. Vienna, Austria: R Foundation for Statistical Computing; 2021.

[54] De Caceres M, Legendre P (2009). Associations between species and groups of sites: indices and statistical inference. Ecology..

[55] Frank E Harrell Jr. wcfCDamo. Hmisc: Harrell Miscellaneous. 2021. R packageversion 4.6-0. https://CRAN.R-project.org/package=Hmisc

[56] Kuhn M, Jackson S, Cimentada J corrr: Correlations in R. 2020. R package version 0.4.3. https://CRAN.R-project.org/package=corrr

[57] Spearman C (1904). The7proof and measurement of association7between two things. Am J Psychol..

[58] Epskamp S, Cramer AOJ, Waldorp LJ, Schmittmann VD, Borsboom D (2012). qgraph: network visualizations of relationships in psychometric data. J Stat Softw..

[59] Kimura M, Jia ZJ, Nakayama N, Asakawa S (2008). Ecology of viruses in soils: Past, present and future perspectives. Soil Sci Plant Nutr..

[60] Williamson KE, Schnitker JB, Radosevich M, Smith DW, Wommack KE (2008). Cultivationbased assessment of lysogeny among soil bacteria. Microb Ecol..

[61] Berns AE, Philipp H, Narres HD, Burauel P, Vereecken H, Tappe W (2008). Effect of gammasterilization and autoclaving on soil organic matter structure as studied by solid state NMR, UV and fluorescence spectroscopy. Eur J Soil Sci..

[62] Tian QX, He HB, Cheng WX, Zhang XD (2014). Pulse-dynamic and monotonic decline patterns of soil respiration in long term laboratory microcosms. Soil Biol Biochem..

[63] Emerson JB, Adams RI, Roman CMB, Brooks B, Coil DA, Dahlhausen K (2017). Schrodinger’s microbes: Tools for distinguishing the living from the dead in microbial ecosystems. Microbiome..

[64] Halgasova N, Ugorcakova J, Gerova M, Timko J, Bukovska G (2010). Isolation and characterization of bacteriophage PhiBP from Paenibacillus polymyxa CCM 7400. FEMS Microbiol Lett..

[65] Klyczek KK, Bonilla JA, Jacobs-Sera D, Adair TL, Afram P, Allen KG (2017). Tales of diversity: Genomic and morphological characteristics of forty-six Arthrobacter phages. PLoS ONE..

[66] Li P, Bhattacharjee P, Wang S, Zhang L, Ahmed I, Guo L (2019). Mycoviruses in fusarium species: an update. Front Cell Infect Microbiol..

[67] Ghabrial SA, Caston JR, Jiang DH, Nibert ML, Suzuki N (2015). 50-plus years of fungal viruses. Virology..

[68] Lopez-Mondejar R, Zuhlke D, Vetrovsky T, Becher D, Riedel K, Baldrian P (2016). Decoding the complete arsenal for cellulose and hemicellulose deconstruction in the highly efficient cellulose decomposer Paenibacillus O199. Biotechnol Biofuels..

[69] Thakur V, Kumar V, Kumar S, Singh D (2018). Diverse culturable bacterial communities with cellulolytic potential revealed from pristine habitat in Indian trans-Himalaya. Can J Microbiol..

[70] Panagiotou G, Kekos D, Macris BJ, Christakopoulos P (2003). Production of cellulolytic and xylanolytic enzymes by Fusarium oxysporum grown on corn stover in solid state fermentation. Ind Crop. Prod..

[71] Zheng HP, Yang TJ, Bao YZ, He PP, Yang KM, Mei XL (2021). Network analysis and subsequent culturing reveal keystone taxa involved in microbial litter decomposition dynamics. Soil Biol Biochem..

[72] Zhou ZH, Wang CK, Zheng MH, Jiang LF, Luo YQ (2017). Patterns and mechanisms of responses by soil microbial communities to nitrogen addition. Soil Biol Biochem..

[73] Peters BM, Jabra-Rizk MA, O’May GA, Costerton JW, Shirtliff ME (2012). Polymicrobial interactions: impact on pathogenesis and human disease. Clin Microbiol Rev..

[74] Carreira C, Lonborg C, Kuhl M, Lillebo AI, Sandaa RA, Villanueva L (2020). Fungi and viruses as important players in microbial mats. Fems Microbiol Ecol..

[75] Hurwitz BL, Hallam SJ, Sullivan MB (2013). Metabolic reprogramming by viruses in the sunlit and dark ocean. Genome Biol..

[76] Sieradzki ET, Ignacio-Espinoza JC, Needham DM, Fichot EB, Fuhrman JA (2019). Dynamic marine viral infections and major contribution to photosynthetic processes shown by spatiotemporal picoplankton metatranscriptomes. Nat Commun..

