# Supplementary material for: Experimental evidence for the impact of soil viruses on carbon cycling during surface plant litter decomposition
Source: ISME Commun. 2022 Mar 16;2:24. doi: 10.1038/s43705-022-00109-4 (PMC9723558; doi:10.1038/s43705-022-00109-4)
Supplement: Supplementary file 5 — Supplementary Figure Legends [file 43705_2022_109_MOESM5_ESM.docx]

**Supplementary Figure Legends**

**Figure S1.** Experimental design. Treatments for each soil type included 1) whole microbial community (no added virus), 2) whole microbial community and killed virus concentrate (+killed-virus), and 3) whole microbial community and virus concentrate (+virus). Whole communities and viral concentrates were mixed together and added to microcosms containing sterilized plant litter on sand.

**Figure S2.** Phage extraction efficiency. A) Amount of lamda phage spiked into the soils (left bar), and the amount of lambda phage that was re-extracted from the PJ and SF soils. NS indicates no significant differences in lambda phage between the positive control (lambda) and the samples. B) Photo of plaque assays showing the *E.coli* + lambda positive control (green), the *E.coli* + lambda extractions from soils (light blue) and the *E.coli* only and *E.coli* + killed lambda negative controls (red).

**Figure S3.** Dissolved organic carbon (DOC) and total nitrogen (TN) in microcosms inoculated with the PJ soil (left) and SF soil (right) after 40 days grouped by treatment, +virus (blue), +killed-virus (red), control (gray). Triangles show estimates of the initial concentration of available DOC and TN in the microcosms. This was calculated by adding DOC/TN concentrations of the initial litter substrate and the inoculum which included the PJ and SF virus, +virus and +virus-killed concentrates, and the M9 Media for the no added virus controls. Letters indicate significant differences in final DOC and TN abundance across treatments and soil inoculum (Tukey hsd posthoc test).

**Figure S4.** Mean relative abundance of A) bacterial and B) fungal taxa showing significant differences between the no added virus compared to the +killed-virus and +virus treatments identified through indicator species analysis. Taxa that were more abundant in the control treatment in both PJ and SF microcosms are highlighted in red.

**Figure S5.** Bacterial and fungal richness and Shannon diversity across treatments for the PJ and SF microcosms. Letters indicate significant differences across treatments and soil inoculum (Tukey hsd posthoc test).

**Figure S6.** Correlations between carbon, nutrient, and microbial community traits across the three treatments for both soils combined. All spearman correlations are shown; correlations with p-values <0.05, 0.01, and 0.001 are denoted with a *,**, and *** respectively.
