# Supplementary figures and images for: Experimental evidence for the impact of soil viruses on carbon cycling during surface plant litter decomposition
Source: ISME Commun. 2022 Mar 16;2:24. doi: 10.1038/s43705-022-00109-4 (PMC9723558; doi:10.1038/s43705-022-00109-4)

A.

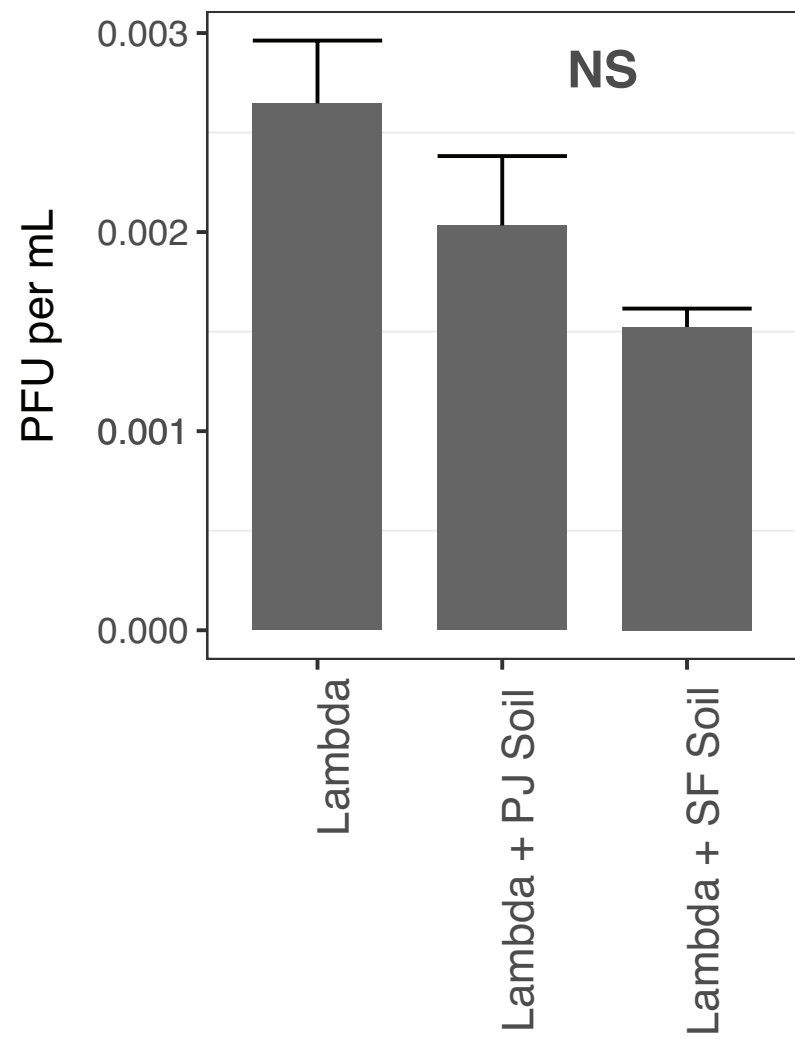

B.

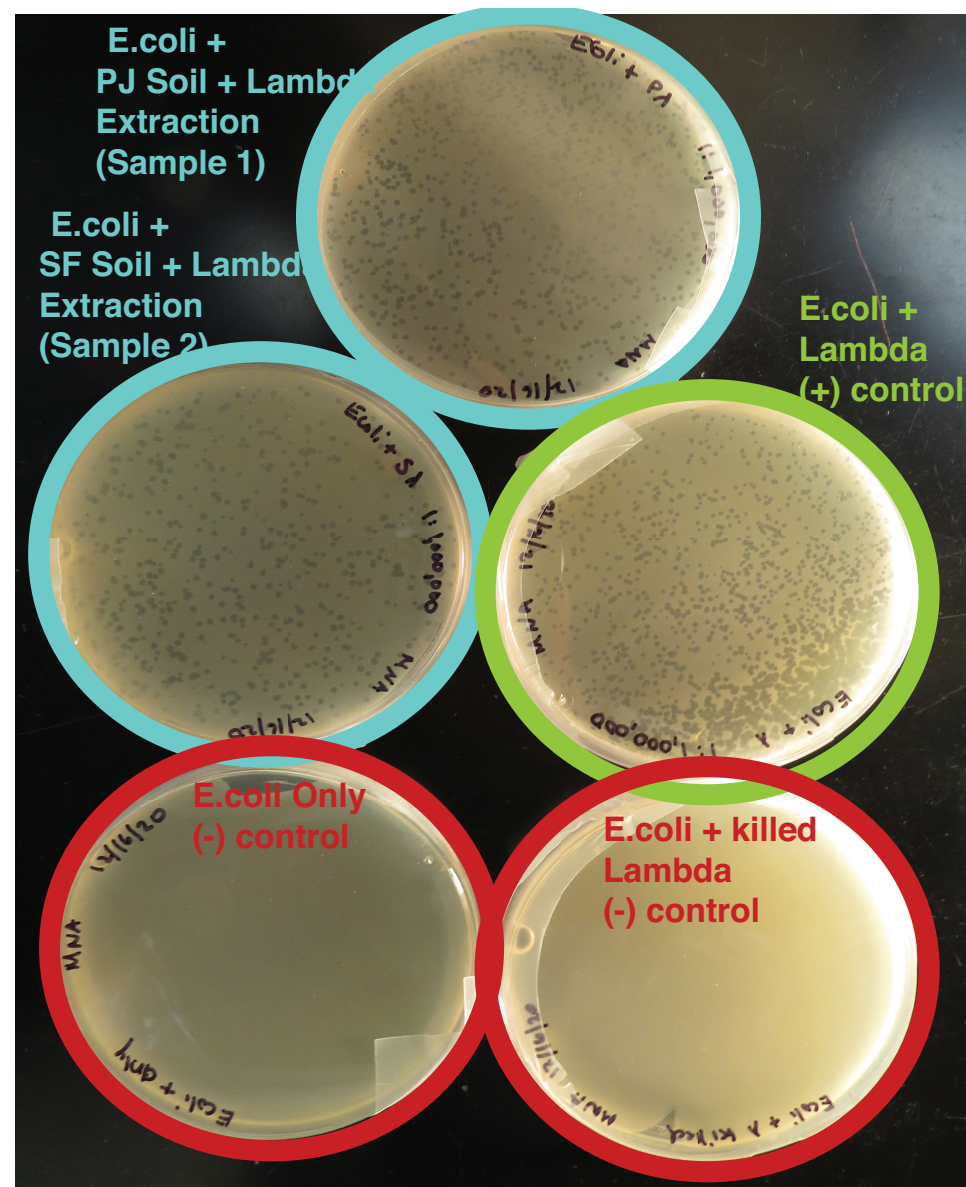

Supplement: Supplementary file 7 — Figure S2 [file 43705_2022_109_MOESM7_ESM.pdf]
